# Glioblastoma: An Update in Pathology, Molecular Mechanisms and Biomarkers

**DOI:** 10.3390/ijms25053040

**Published:** 2024-03-06

**Authors:** Zhong Lan, Xin Li, Xiaoqin Zhang

**Affiliations:** Department of Pathology, School of Medicine, South China University of Technology, Guangzhou 510006, China; 202030520118@mail.scut.edu.cn (Z.L.); 202130520162@mail.scut.edu.cn (X.L.)

**Keywords:** glioblastoma, histopathology, molecular pathology, genetics, epigenetics, transcriptomics, signaling pathway, biomarkers

## Abstract

Glioblastoma multiforme (GBM) is the most common and malignant type of primary brain tumor in adults. Despite important advances in understanding the molecular pathogenesis and biology of this tumor in the past decade, the prognosis for GBM patients remains poor. GBM is characterized by aggressive biological behavior and high degrees of inter-tumor and intra-tumor heterogeneity. Increased understanding of the molecular and cellular heterogeneity of GBM may not only help more accurately define specific subgroups for precise diagnosis but also lay the groundwork for the successful implementation of targeted therapy. Herein, we systematically review the key achievements in the understanding of GBM molecular pathogenesis, mechanisms, and biomarkers in the past decade. We discuss the advances in the molecular pathology of GBM, including genetics, epigenetics, transcriptomics, and signaling pathways. We also review the molecular biomarkers that have potential clinical roles. Finally, new strategies, current challenges, and future directions for discovering new biomarkers and therapeutic targets for GBM will be discussed.

## 1. Introduction

Glioblastoma multiforme (GBM) is the most common type of malignant primary brain tumor, accounting for over half of all primary malignant tumors in the Central Nervous System (CNS) [1,2,3]. GBM is categorized as grade 4 (most malignant) astrocytic glioma in the World Health Organization (WHO) classification of brain tumors [4]. It is highly aggressive and fast-growing and often diffusely invades the surrounding brain tissues, which makes it the deadliest form of tumor in the brain [5,6]. The current standard treatment regimen for GBM is surgical resection followed by radiotherapy plus concomitant and adjuvant chemotherapy with temozolomide [7]. Despite the advances in surgery, radiation, and chemotherapy in the past decade, the overall survival of GBM patients remains poor, with a median survival of only 12–15 months [5,7,8,9].

Increasingly, studies have shown that GBM is a highly heterogeneous group of tumors, and the pathogenesis of GBM involves complex alterations in genetics, epigenetics, and transcriptomics, which finally lead to significant changes in major signaling pathways [10,11,12,13,14,15,16,17]. The characterization of molecular features of GBM, therefore, may not only help provide a better understanding of tumor pathogenesis but also help prognostication and assist in making decisions in targeted therapy [13,18,19,20,21,22]. Since the update of the 4th edition of the WHO classification of CNS tumors (CNS 4) published in 2016 [23], molecular markers have been listed as part of the classification of brain tumors, including GBM, emphasizing the importance of molecular biomarkers in GBM pathological diagnosis. Together with the new advances in technology or concept of GBM biological research, e.g., single-cell technology [24,25,26,27], deep learning-based multi-omics data exploration [28,29,30], novel 3D preclinical GBM models [31,32,33], and emphasis on tumor microenvironment [34,35,36], which have shed new lights for the molecular characterization of GBM, there is a need for an updated and integrated overview of the molecular underpinnings of GBM and their potential clinical applicability.

Here, we aim to provide an update on the progress of GBM pathology, molecular mechanisms, and biomarkers achieved so far. We will comprehensively review the molecular features of GBM at multimodal levels, including genetics, epigenetics, and transcriptomics, and how they integrate with histopathological features and relate to patient diagnosis, prognosis, and treatment. We will also summarize the obstacles and challenges in implementing molecular features in the diagnostics, prognostication, and therapeutics of GBM. Finally, new strategies and future directions for the development of new biomarkers with clinical potential are discussed.

## 2. Histopathology of GBM

**Figure 1:**
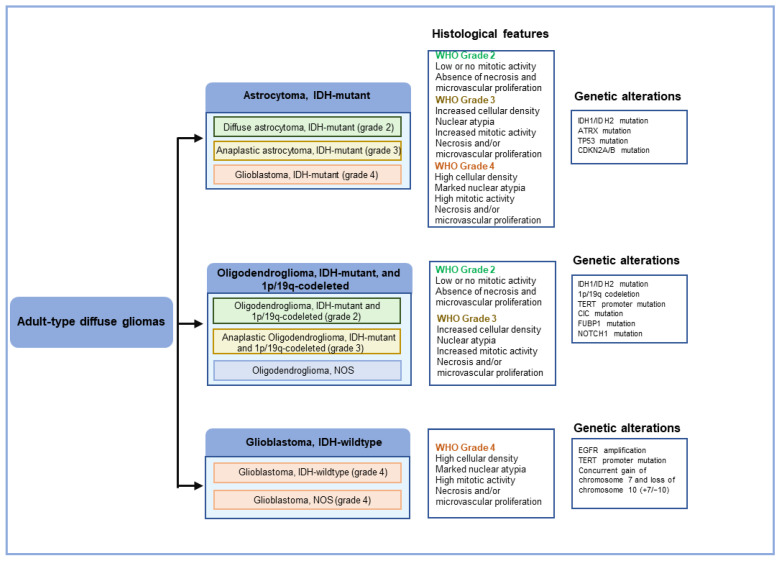
Integrated histological and molecular classification of the major diffuse gliomas by WHO.

The traditional diagnosis of GBM is largely based on its histopathological features. According to the guidelines of the World Health Organization (WHO) for the classification of Central Nervous System (CNS) tumors, gliomas can be divided into two major categories based on the degree of invasiveness into the surrounding brain tissue: diffuse gliomas and circumscribed gliomas (Table 1; Figure 1) [4,23]. Diffuse gliomas have the ability to infiltrate surrounding normal brain parenchyma and, unfortunately, inevitably recur even after gross total resection. Circumscribed gliomas, in contrast, have well-defined margins and are generally benign. Diffuse gliomas occur more commonly than circumscribed gliomas and are the most common intrinsic primary brain tumors. Based on malignancy grade, diffuse gliomas are divided into three grades: WHO grades 2, 3, and 4, with WHO grade 4 diffuse glioma being synonymous with GBM. Based on histologic entities, diffuse gliomas can be astrocytic or oligodendroglial. The most common histologic subtype of diffuse gliomas is astrocytoma with a WHO grade 4, that is, GBM, which accounts for ~50% of all primary malignant brain tumors.

The classic morphologic features of GBM include nuclear atypia, cellular pleomorphism, mitotic activity, microvascular proliferation, and (or) necrosis. GBM also has several uncommon variants, including gliosarcomas, which display high-grade, malignant astrocytic features and also contain prominent sarcoma-like mesenchymal metaplasia elements; giant-cell glioblastomas, which have large, highly pleomorphic, multinucleated giant cells; small-cell glioblastomas, which are associated with amplification of the epidermal growth factor receptor (EGFR); glioblastomas with oligodendroglial features, which may be associated with a better prognosis than standard glioblastomas; and finally, epithelioid glioblastomas, a newly accepted variant, which is characterized by prominent epithelioid morphology and high proportion of BRAF V600E mutations in tumor cells [37].

GBM can be further separated into two major classes: primary GBM and secondary GBM, with the majority being primary [5,6,7]. Primary GBM arises de novo with no known clinical precursor, and most occur in elderly adults (older than 50 years of age), while secondary GBM is a result of progression from a pre-existing lower malignancy grade and usually affects younger patients. Primary and secondary GBM are morphologically indistinguishable and respond similarly to conventional therapy, but they have different molecular features and, therefore, may respond differently to targeted molecular therapies [10].

## 3. Molecular Pathology of GBM

While the above histopathology-based morphologic classification provides important information for the diagnosis of GBM, it has a limitation in that it cannot reflect the heterogeneity of GBM tumors and, therefore, is insufficient for patient management. The 2016 revision of the WHO classification of CNS tumors (CNS 4), therefore, restructured the classification of GBM by incorporating molecular features into the histopathologic appearances. For example, for the diagnosis of GBM, IDH mutation status was included to classify patients into distinct subgroups, namely, glioblastoma, IDH–wild-type and glioblastoma, IDH-mutant type. IDH–wild-type glioblastoma corresponds to the clinically defined primary glioblastoma characterized by de novo development with no identifiable precursor lesion. This cohort represents the overwhelming majority of patients with glioblastoma (~90%), is more commonly diagnosed in older patients, and has a more aggressive clinical course. Conversely, IDH-mutant glioblastoma or secondary glioblastoma typically arises from a precursor diffuse or anaplastic astrocytoma. This cohort represents approximately 10% of patients and predominates in younger patients with a median age at diagnosis of 44 years, which generally carries a better prognosis.

This shift toward molecular classification of primary brain tumors is further emphasized in the 2021 revision of the WHO classification of CNS tumors (CNS 5), which incorporates more molecular characteristics as part of the definition of gliomas (Table 2; Figure 1) [4]. These include CDKN2A/B homozygous deletion mutation, TERT promoter mutation, EGFR gene amplification, and combined gain of entire chromosome 7 and loss of entire chromosome (+7/−10) as qualifying for the diagnosis of GBM, IDH-wildtype (Table 2) [4]. In doing so, WHO CNS 5 advances the role of molecular diagnostics in GBM sub-classification.

In addition to the above-mentioned molecular biomarkers that have been integrated into WHO classification, there is substantial evidence for other molecular changes characterized in GBM. These studies have not only revealed the molecular heterogeneity of GBM at multiple genome-wide levels but also provided useful insights into the fundamental mechanisms for the pathogenesis of GBM [10,11,12,13,14,15,16,17]. In the following, we will discuss the major advances in molecular pathology of GBM at each molecular level, including genetics, epigenetics, and transcriptomics, and how these advances may help refine GBM classification into distinct sub-groups with important clinical implications for future studies.

### 3.1. Genetic Changes in GBM

The key genetic alterations characterized in GBM include TERT promoter mutation, PTEN tumor suppressor gene deletion, high-level gene amplification of proto-oncogene EGFR, ATRX mutation, and TP53 mutation [4,17,21]. Amongst, TERT promoter mutation, PTEN deletion, and EGFR amplification are more frequently present in primary GBM (IDH wild-type GBM), while ATRX mutation and TP53 mutation are much more common in secondary GBM (IDH mutant-type GBM) [21]. Other important genetic alterations reported in GBM include NF1, PDGFRA, PIK3R1, PIK3CA, RB1, CDKN2A/B, MDM2, MDM4, CDK4, and H3F3A [13,15,16,18]. Generally, the genetic abnormalities in GBM are characterized by three major biological processes: initiating tumor growth, evading senescence, and enabling immortal growth [5,21]. Genetic defects in each of these three processes seem required for gliomagenesis through the key signaling pathways.

### 3.2. Epigenetic Changes in GBM

Epigenetic modifications, including DNA methylation, histone modification, and chromatin remodeling, have been regarded as a hallmark of GBM tumorigenesis and development [38,39,40]. They were not only implicated as potential biomarkers for optimal clinical patient stratification but also potential drug targets because of their reversibility [38,39,40]. One of the best-studied examples is MGMT promoter methylation status, which was found to predict the benefit of alkylating chemotherapy and be of clinical importance in GBM patient prognostication [41,42]. Inspired by this finding, epigenetic alterations have been extensively characterized at a genome-wide scale in GBM and revealed some interesting insights [43]. For example, by profiling promoter DNA methylation alterations in 272 GBM tumors from The Cancer Genome Atlas (TCGA), an international joint program to systematically explore the genomic changes involved in human cancer, a distinct subset of samples with concerted hypermethylation at a large number of CpG Island loci (G-CIMP) have been identified [44]. Moreover, the patients with the G-CIMP phenotype demonstrated distinct clinical features and prognoses, indicating the potential role of epigenetic alteration in refining patient classification [44]. Similarly, a DNA methylation-based profile could classify CNS tumors (including GBM) into different cancer entities, indicating again the potential of epigenetic alterations in cancer diagnosis [45]. It was even found that the DNA methylation landscape demonstrated extensive heterogeneity in time and space during GBM progression [46]. More recently, epigenetic remodeling was also linked with tumor microenvironment (TME) in that it could affect the immune cell activity and modulate antitumor immune response within the TME of GBM [47].

Therapeutically, epigenetic modulators have shown promising results in various cancers, including GBM, and have been investigated in clinical trials as antitumor agents [39,40,47,48]. For example, histone deacetylase (HDAC) inhibitor (Vorinostat) has been tested in a Phase II study for patients with recurrent GBM [49]. Inhibitors of histone methyltransferases, e.g., PRMT5, could induce cell apoptosis and drive undifferentiated primary patient-mediated GBM cells into a non-replicative senescence state both in vitro and in vivo studies, suggesting its potential as a druggable target for GBM therapy [50]. Mechanistically, epigenetic alterations could lead to transcriptional aberrations and affect various biological processes, including cell cycle, cell differentiation, angiogenesis, and apoptosis, and ultimately regulate the proliferation and growth of tumor cells of GBM [39,48]. A comprehensive overview of the preclinical and clinical studies on epigenetic modulators as therapeutic agents for GBM can be found in several recent good review papers [38,39,40,48].

### 3.3. Transcriptomic Changes in GBM

The transcriptomic features of GBM have been extensively studied with the advance of high-throughput transcriptome profiling methods and computational analysis tools. Now, it is widely accepted that GBM is a heterogenous tumor with widespread transcriptional heterogeneity [10,11,12,13,14,15,16,17]. In fact, GBM is one of the early tumor types that have been systemically investigated by large international cancer projects, e.g., TCGA. For example, based on the microarray profiling expression platform, Phillips et al. clustered GBM samples into three distinct subtypes: Proneural, Proliferative, and Mesenchymal, which differ significantly in patient prognosis [51]. Following that, Verhaak et al. expanded the work to a larger TCGA GBM sample cohort and identified four major subtypes in GBM: Proneural, Proliferative (or Classical), Mesenchymal, and Neural [52]. This study confirmed the findings of prior work and validated the robustness of transcriptome-based molecular classification in GBM. These works provide a strong rationale for the use of transcriptome-based subtyping to refine histopathology-based GBM classification to provide better prognostic prediction. More importantly, they provide associations between transcriptomic changes and other molecular alterations (e.g., genetic and epigenetic alteration, detailed below), which shed light on the fundamental mechanisms of tumorigenesis and progression of GBM.

While the above-mentioned studies provide valuable insights into the transcriptomic alteration in GBM pathogenesis and classification, they have limitations in that these findings are derived from the bulk tumor tissue and, therefore, cannot address the intra-tumor heterogeneity, which is pervasive in GBM [24,53], and also the confounding effect of tumor microenvironment, which also plays important role in regulating GBM biological behavior as demonstrated by increasing more recent studies [34,54,55]. These limitations are corrected by recently emerging studies at single-cell resolution [24,56]. By means of the advancement of sequencing technology, single-cell RNA sequencing (scRNA-seq) has begun to uncover the hidden composition of complex tumor ecosystems in GBM [26,57,58]. It has become clear that GBM heterogeneity not only lies within the heterogeneity of the tumor but also its associated microenvironment and strongly differs between new and recurrent GBM, which has been designated as the main cause of treatment failure [26,57,58,59,60,61,62,63]. While a detailed review of these scRNA-seq-based studies is beyond the scope of the current paper, readers are encouraged to review the original readings listed in the references [26,57,58,59,60,61,62,63].

### 3.4. Correlation between Genetic, Epigenetic and Transcriptomic Alterations in GBM

The molecular abnormalities characterized at each dimension (genetics, epigenetics, and transcriptomics) are not isolated but correlate with each other. For example, among the four transcriptomics-based GBM subtypes (Proneural, Proliferative, Mesenchymal, and Neural, the Proneural subtype was found to be enriched with PDGFR amplification, TP53 mutation, and IDH1 mutation; the Proliferative subtype demonstrated a greater preponderance of EGFR amplification, decreased rates of TP53 mutation, along with p16INK4A and p14ARF deletion; the Mesenchymal subtype was found to have a greater degree of NF1 mutation, along with alterations of PTEN and Akt; the Neural subtype was found to have a greater degree of neuronal marker expression and the histology was consistent with a combination of oligodendroglial, astrocytic, and neuronal features [52]. For the correlation between epigenetic and transcriptomic alterations, it was found that the G-CIMP epigenetic phenotype GBM tumor, which displayed hypermethylation at a large number of CpG island loci [44], was more associated with Proneural subtype [52]. In the pediatric GBM, among the three tumor subtypes classified by DNA methylation landscape (MYCN, RTK1, and RTK2), it was found that MYCN was enriched for MYCN amplification, RTK1 enriched for PDGFRA amplification, and RTK 2 enriched for EGFR amplification [64]. Another integrative study spanning genetics, transcriptomics, and functional approaches revealed four cellular states within GBM tumor and demonstrated that the cellular states plasticity was influenced by copy number amplifications of the CDK4, EGFR, and PDGFRA and by mutations of NF1, which each favor a defined state [63]. The correlation between molecular alterations at different dimensional levels provides important insights into the mechanism underlying GBM pathogenesis and progression and deserves future studies.

### 3.5. Key Signaling Pathways Altered in GBM

**Figure 2:**
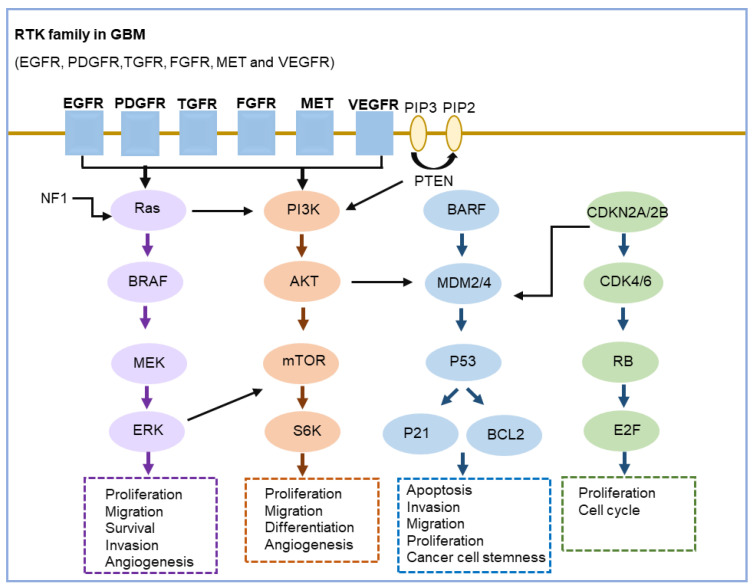
Schematic representation of the key signaling pathways altered in GBM.

While the above studies have provided useful insights into the molecular pathogenesis of GBM, they have also revealed the key signaling pathways involved in tumorigenesis and progression of GBM. There are three key signaling pathways that are consistently and commonly altered in GBM [11,12,13,19,21,65]: (1) RTK pathway, (2) TP53 pathway, and (3) RB pathway [65] (Figure 2). These pathways have some degree of overlap and interact with each other; some genes that are involved in one pathway may also play roles in other pathways (Figure 2).

***(1) RTK pathway.*** RTK (receptor tyrosine kinase) signaling is the most frequently altered signaling pathway in GBM, especially in IDH-wildtype GBM tumors. RTK is a cell-surface receptor that binds growth factors [66], the family of which includes EGFR, PDGFR, TGFR, FGFR, MET, and VEGFR, and is an essential component of signal transduction pathways that mediate cell-to-cell communication. In GBM, the activation of RTK signaling through the PI3K/AKT/mTOR pathway induces cell proliferation, migration, differentiation, and survival [67,68]. The most common targets of the RTK pathway are EGFR and PTEN, the former acting in an oncogenic role while the latter acting as a tumor suppressor. In GBM cells, the activation of EGFR and the PI3K/AKT/mTOR signaling could be achieved either through amplification of the EGFR (resulting in overexpression of EGFR) and/or EGFR mutation [69]. The negative regulator of the pathway, PTEN, could be inactivated through mutation or deletion, and thus facilitates the pathway activation and induces cell migration, invasion, and survival. Another commonly altered RTK pathway in GBM is the Ras pathway (Ras/BRAF/MEK) [67,68]. Active Ras (Ras-GTP) promotes cell cycle progression, cell survival, and migration through a cascade of downstream effectors [67,68]. RTK has been suggested as a druggable target in GBM and is extensively investigated in clinical trials [68,70,71,72].

***(2) TP53 pathway.*** TP53 is a well-known tumor suppressor and transcription factor gene, which plays critical roles in tumor prevention by regulating a wide variety of cellular processes, including invasion, migration, proliferation, evasion of apoptosis, and cancer cell stemness. The TP53/MDM2/CDKN2A pathway is deregulated in 84% of GBM patients and 94% of GBM cell lines [73]. Inactivation of TP53 by mutation, which is found in ~1/3 IDH-wildtype GBM and 2/3 IDH-mutant GBM, leads to the loss of its tumor suppressive functions and, therefore, tumorigenesis. MDM2 is an inhibitor of p53, mediating p53 degradation and thereby promoting tumorigenesis. MDM2 amplification is usually mutually exclusive with the TP53 mutation and is more frequently found in primary GBMs that lack the TP53 mutation. CDKN2A is another important regulator of the TP53 pathway and the homozygous deletion of CDKN2A, which is prevalent in 22–35% of all GBMs (16–47% IDH-mutant GBM and ~58% of IDH-wildtype GBM), leads to inactivation of TP53 pathway and is associated with lower overall survival of GBM patients [65,73]. CDKN2A has currently been incorporated into the WHO classification of glioma [4], suggesting its important potential as a landmark marker in GBM clinical management.

***(3) RB pathway.*** The retinoblastoma protein (RB) pathway is also found to be frequently altered in GBM and plays a crucial role in regulating tumorigenesis in GBM [65,68]. The phosphorylation of RB protein, which is accomplished by the CDK4/Cyclin D1 complex, can inhibit the cell cycle progress from the G1 to S phase by binding with the E2F transcription factor. RB pathway could be joined with the TP53 pathway through CDKN2A, which encodes Ink4a and Arf proteins and plays an important role in activating RB and TP53, respectively. The growth inhibition function of the RB pathway is often disrupted in GBM, most commonly due to inactivation of CDKN2A/CDKN2B and RB1 and amplification of CDK4 and CDK6 [74,75]. Methylation of the RB1 promoter, which is frequent in secondary GBM (IDH-mutant ones), can also result in decreased RB1 expression and cell-cycle checkpoint function and finally leads to dysregulated cell cycle and uncontrolled cell proliferation. CDK4 and CDK6 inhibitors have shown promising antitumor efficacy in GBM and are being studied in clinical trials [76].

## 4. Clinically Relevant Molecular Biomarkers in GBM

Among the abundant molecular biomarkers identified above, three of them have demonstrated the greatest potential in the clinical practice of GBM, including IDH1, MGMT, and EGFR [42,77,78,79,80,81]. IDHI mutation has been widely shown to be associated with better prognosis of GBM patients [79,80,82]. IDH1 mutations are typically found in younger patients (secondary GBM) that have high frequencies of TP53 mutations. They have been incorporated into WHO diagnosis guidelines for GBM and used as a positive predictor of prognosis [4]. MGMT promoter methylation is also one of the most relevant prognostic markers in GBM, although it is not included in the current WHO classification guideline as IDH1 mutation status. It can be used to predict therapeutic response to alkylating agents such as temozolomide [41,42,81]. Silencing MGMT by promoter methylation would lead to enhanced cytotoxic activity of temozolomide and thus increased patient survival. EGFR abnormality, which is driven by amplification and/or EGFRvIII mutation, is a prognostic marker in GBM and correlates with higher tumor malignancy, poorer prognosis, and shorter survival time [77,78]. In addition to its prognostic role, EGFR can also be employed as a therapeutic target. Anti-EGFR therapy, tyrosine kinase inhibitors, such as Gefitinib and Erlotinib, have been tested in clinical trials to block the downstream signaling of EGFR by preventing phosphorylation of tyrosine residues [70,71,72,83,84].

Numerous new biomarkers are being tested in clinical trials. These generally include growth factor receptor inhibitors [85,86,87,88], angiogenesis inhibitors [89,90], and miscellaneous agents (immunotherapies and therapies targeting tumor cell metabolism [91,92,93,94]. Growth factor receptor inhibitors are currently being studied through the strategy of targeting stem cells and stem cell pathways, targeting cell growth autonomy and migration, targeting cell cycle, and escape to cell death. The best-studied example is anti-EGFR agents, more generally tyrosine kinase inhibitors (TKIs). A series of TKIs, including Erlotinib, Gefitinib, and Afatinib, have been investigated in several Phase II or III studies [77,85,95]. Targeting angiogenesis by angiogenesis inhibitors is another research direction due to the highly angiogenic nature of GBM tumors. Clinical trials for targeting the VEGF/VEGFR pathway (e.g., bevacizumab) have been approved by the Food and Drug Administration (FDA) since 2009 as a treatment of recurrent GBM [89,90]. For immunotherapy, innovative immune-targeting strategies, including cancer vaccines, oncolytic viruses, checkpoint blockade inhibitors, adoptive cell transfer, and CAR T cells, have been investigated in GBM [96,97,98,99].

## 5. Challenges and Future Directions

While great strides have been made in the molecular characterization of GBM, there are still many challenges in the implementation of these discoveries into clinical management. This can be exemplified by the fact that the survival rates of GBM patients have remained relatively unchanged since the introduction of the Stupp protocol in 2005. One of the biggest challenges is intratumor heterogeneity, which poses a major obstacle to the treatment failure of GBM. It is increasingly accepted that there is spatial heterogeneity within the same tumor. For example, some tumor regions are hypoxic and necrotic, and others are more normoxic; some regions are more proliferative, with others very quiescent; some regions are more vascularized, whereas some are more infiltrative. These phenotypic features are also accompanied by genotypic differences, which have been demonstrated by recent single-cell-based molecular analyses. This intratumor heterogeneity and inherent molecular complexity of GBM will, therefore, necessitate combination therapy in the future by employing multimodal agents to co-target the diverse driver events instead of the current single-target strategy. The second key challenge is the pharmacodynamic and pharmacokinetic failure. Poor drug distribution within the brain because of the natural exclusion of blood–brain barrier (BBB) makes adequate drug delivery a critical challenge in GBM treatment. For example, EGFR inhibitor lapatinib has been shown to inhibit EGFRvIII in vitro by preferentially binding the inactive conformation of the kinase but fails to achieve sufficient intratumor concentrations in GBM patients. These findings reinforce the need to consider BBB penetration of the selected therapy during therapeutic planning.

Given these challenges, a more rational design of both lab research and clinical trial is needed in the future study. At the lab research level, it is important to obtain patient biopsies from multiple tumor regions with different infiltrative characteristics and perform comprehensive genome-wide molecular studies to select the therapeutic targets. During the target selection, multiple agents and combinational therapy are recommended according to the molecular profiles of the patient tumor sample. Drug pharmacokinetics and drug-to-tumor delivery strategies to ensure biologically adequate distribution and pharmacodynamic changes within brain and GBM tumors also need to be considered. Finally, whenever possible, patients’ blood samples should be obtained over time to assess for circulating molecules that may help with noninvasive biomarker development in the future.

It is important to note that although this review tries its best to comprehensively and systematically summarize the molecular study of GBM achieved so far, some relevant reports may have been missed. Moreover, the quality of the included studies was not assessed, and the review is limited by the quality of the evidence.

## 6. Summary

Taken together, great progress has been made in the molecular characterization of GBM in the past decade. While these studies provide useful insights into the fundamental mechanisms of GBM pathogenesis, there are still many challenges in the implementation of these discoveries into clinical management. With continued efforts in higher resolution molecular subtype signatures of GBM, combined with gene therapy, immunotherapy, and organoid technology, the concept of precision medicine is expected to be achieved in GBM in the future.

## Figures and Tables

**Table 1:** Glioma classification according to World Health Organization (WHO) 2021.

Tumor Type	CNS WHO Grade
**Adult-type diffuse gliomas**	
Astrocytoma, IDH-mutant	2, 3, 4
Oligodendroglioma, IDH-mutant, and 1p/19q-codeleted	2, 3
Glioblastoma, IDH-wildtype	4
**Pediatric-type diffuse low-grade gliomas**	
Diffuse astrocytoma, MYB- or MYBL1-altered ^#^	1
Angiocentric glioma	1
Polymorphous low-grade neuroepithelial tumor of the young ^#^	1
Diffuse low-grade glioma, MAPK pathway-altered *^#^	-
**Pediatric-type diffuse high-grade gliomas**	
Diffuse midline glioma, H3 K27-altered	4
Diffuse hemispheric glioma, H3 G34-mutant ^#^	4
Diffuse pediatric-type high-grade glioma, H3-wildtype and IDH-wildtype ^#^	4
Infant-type hemispheric glioma *^#^	-
**Circumscribed astrocytic gliomas**	
Pilocytic astrocytoma	1
High-grade astrocytoma with piloid features *^#^	-
Pleomorphic xanthoastrocytoma	2, 3
Subependymal giant cell astrocytoma	1
Chordoid glioma	2
Astroblastoma, MN1 altered *	-
**Ependymal tumors**	

^#^ Newly recognized tumor types in 2021 WHO classification of CNS tumors. * Definitive CNS WHO grade not established.

**Table 2:** Key diagnostic genetic alterations in glioma.

Tumor Type	Genes/Molecular Profiles Characteristically Altered
Astrocytoma, IDH-mutant	IDH1, IDH2, ATRX, TP53, CDKN2A/B
Oligodendroglioma, IDH-mutant, and 1p/19q-codeleted	IDH1, IDH2, 1p/19q, TERT promoter, CIC, FUBP1, NOTCH1
Glioblastoma, IDH-wildtype	IDH-wild type, TERT promoter, chromosomes 7/10, EGFR
Diffuse astrocytoma, MYB- or MYBL1-altered	MYB, MYBL1
Angiocentric glioma	MYB
Polymorphous low-grade neuroepithelial tumor of the young	BRAF, FGFR family
Diffuse low-grade glioma, MAPK pathway-altered	FGFR1, BRAF
Diffuse midline glioma, H3 K27-altered	H3 K27, TP53, ACVR1, PDGFRA, EGFR, EZHIP
Diffuse hemispheric glioma, H3 G34-mutant	H3 G34, TP53, ARTX
Diffuse pediatric-type high-grade glioma, H3-wildtype, and IDH-wildtype	IDH-wildtype, H3-wildtype, PDGFRA, MYCN, EGFR (methylome)
Infant-type hemispheric glioma	NTRK family, ALK, ROS, MET
Pilocytic astrocytoma	KIAA1549-BRAF, BRAF, NF1
High-grade astrocytoma with piloid features	BRAF, NF1, ATRX, CDKN2A/B (methylome)
Pleomorphic xanthoastrocytoma	BRAF, CDKN2A/B
Subependymal giant cell astrocytoma	TSC1, TSC2
Chordoid glioma	PRKCA
Astroblastoma, MN1-altered	MN1
Supratentorial ependymomas	ZFTA, RELA, YAP1, MAML2
Posterior fossa ependymomas	H3 K27me3, EZHIP (methylome)
Spinal ependymomas	NF2, MYCN

The table is modified from Louis et al. Neuro-Oncology. The 2021 WHO Classification of Tumors of the Central Nervous System: A summary [4].

## Abbreviations

GBM, glioblastoma multiforme; CNS, Central Nervous System; WHO, World Health Organization; TME, tumor microenvironment; TCGA, The Cancer Genome Atlas; G-CIMP, glioblastoma CpG Island methylator phenotype; scRNA-seq, single-cell RNA sequencing; RTK, receptor tyrosine kinase; TKIs, tyrosine kinase inhibitors; FDA, Food and Drug Administration; BBB, Blood–Brain Barrier.

## References

[1] Miller K.D., Ostrom Q.T., Kruchko C., Patil N., Tihan T., Cioffi G., Fuchs H.E., Waite K.A., Jemal A., Siegel R.L. (2021). Brain and other central nervous system tumor statistics, 2021. CA Cancer J. Clin..

[2] Xiao D., Yan C., Li D., Xi T., Liu X., Zhu D., Huang G., Xu J., He Z., Wu A. (2023). National Brain Tumour Registry of China (NBTRC) statistical report of primary brain tumours diagnosed in China in years 2019–2020. Lancet Reg. Health. West. Pac..

[3] Ostrom Q.T., Price M., Neff C., Cioffi G., Waite K.A., Kruchko C., Barnholtz-Sloan J.S. (2023). CBTRUS Statistical Report: Primary Brain and Other Central Nervous System Tumors Diagnosed in the United States in 2016–2020. Neuro-Oncology.

[4] Louis D.N., Perry A., Wesseling P., Brat D.J., Cree I.A., Figarella-Branger D., Hawkins C., Ng H.K., Pfister S.M., Reifenberger G. (2021). The 2021 WHO Classification of Tumors of the Central Nervous System: A summary. Neuro-Oncology.

[5] Wen P.Y., Weller M., Lee E.Q., Alexander B.M., Barnholtz-Sloan J.S., Barthel F.P., Batchelor T.T., Bindra R.S., Chang S.M., Chiocca E.A. (2020). Glioblastoma in adults: A Society for Neuro-Oncology (SNO) and European Society of Neuro-Oncology (EANO) consensus review on current management and future directions. Neuro-Oncology.

[6] Oronsky B., Reid T.R., Oronsky A., Sandhu N., Knox S.J. (2021). A Review of Newly Diagnosed Glioblastoma. Front. Oncol..

[7] Tan A.C., Ashley D.M., Lopez G.Y., Malinzak M., Friedman H.S., Khasraw M. (2020). Management of glioblastoma: State of the art and future directions. CA Cancer J. Clin..

[8] Aldape K., Brindle K.M., Chesler L., Chopra R., Gajjar A., Gilbert M.R., Gottardo N., Gutmann D.H., Hargrave D., Holland E.C. (2019). Challenges to curing primary brain tumours. Nat. Rev. Clin. Oncol..

[9] Schaff L.R., Mellinghoff I.K. (2023). Glioblastoma and Other Primary Brain Malignancies in Adults: A Review. JAMA-J. Am. Med. Assoc..

[10] Molinaro A.M., Taylor J.W., Wiencke J.K., Wrensch M.R. (2019). Genetic and molecular epidemiology of adult diffuse glioma. Nat. Rev. Neurol..

[11] Esemen Y., Awan M., Parwez R., Baig A., Rahman S., Masala I., Franchini S., Giakoumettis D. (2022). Molecular Pathogenesis of Glioblastoma in Adults and Future Perspectives: A Systematic Review. Int. J. Mol. Sci..

[12] Aldape K., Zadeh G., Mansouri S., Reifenberger G., von Deimling A. (2015). Glioblastoma: Pathology, molecular mechanisms and markers. Acta Neuropathol..

[13] Verdugo E., Puerto I., Medina M.A. (2022). An update on the molecular biology of glioblastoma, with clinical implications and progress in its treatment. Cancer Commun..

[14] Liebermana F. (2017). Glioblastoma update: Molecular biology, diagnosis, treatment, response assessment, and translational clinical trials. F1000Research.

[15] Ludwig K., Muthukrishnan S.D., Alvarado A.G., Kornblum H.I. (2021). Overview of glioblastoma biological hallmarks and molecular pathology. Glioblastoma Resistance to Chemotherapy: Molecular Mechanisms and Innovative Reversal Strategies.

[16] D’Alessio A., Proietti G., Sica G., Scicchitano B.M. (2019). Pathological and Molecular Features of Glioblastoma and Its Peritumoral Tissue. Cancers.

[17] Delgado-Martin B., Medina M.A. (2020). Advances in the Knowledge of the Molecular Biology of Glioblastoma and Its Impact in Patient Diagnosis, Stratification, and Treatment. Adv. Sci..

[18] Reifenberger G., Wirsching H.G., Knobbe-Thomsen C.B., Weller M. (2017). Advances in the molecular genetics of gliomas—Implications for classification and therapy. Nat. Rev. Clin. Oncol..

[19] Le Rhun E., Preusser M., Roth P., Reardon D.A., van den Bent M., Wen P., Reifenberger G., Weller M. (2019). Molecular targeted therapy of glioblastoma. Cancer Treat. Rev..

[20] Zhang P., Xia Q., Liu L.Q., Li S.W., Dong L. (2020). Current Opinion on Molecular Characterization for GBM Classification in Guiding Clinical Diagnosis, Prognosis, and Therapy. Front. Mol. Biosci..

[21] del Pilar Guillermo Prieto M., de La Fuente M.I. (2021). The Role of Molecular Genetics of Glioblastoma in the Clinical Setting. Precision Molecular Pathology of Glioblastoma.

[22] Yang K.Y., Wu Z.J., Zhang H., Zhang N., Wu W.T., Wang Z.Y., Dai Z.Y., Zhang X., Zhang L.Y., Peng Y. (2022). Glioma targeted therapy: Insight into future of molecular approaches. Mol. Cancer.

[23] Louis D.N., Perry A., Reifenberger G., von Deimling A., Figarella-Branger D., Cavenee W.K., Ohgaki H., Wiestler O.D., Kleihues P., Ellison D.W. (2016). The 2016 World Health Organization Classification of Tumors of the Central Nervous System: A summary. Acta Neuropathol..

[24] Eisenbarth D., Wang Y.A. (2023). Glioblastoma heterogeneity at single cell resolution. Oncogene.

[25] Johnson K.C., Anderson K.J., Courtois E.T., Barthel F.P., Varn F.S., Luo D.N., Seignon M., Yi E., Kim H., Estecio M.R.H. (2021). Single-cell multimodal glioma analyses identify epigenetic regulators of cellular plasticity and environmental stress response. Nat. Genet..

[26] LeBlanc V.G., Trinh D.L., Aslanpour S., Hughes M., Livingstone D., Jin D., Ahn B.Y., Blough M.D., Cairncross J.G., Chan J.A. (2022). Single-cell landscapes of primary glioblastomas and matched explants and cell lines show variable retention of inter- and intratumor heterogeneity. Cancer Cell.

[27] Wang L., Jung J., Babikir H., Shamardani K., Jain S., Feng X., Gupta N., Rosi S., Chang S., Raleigh D. (2022). A single-cell atlas of glioblastoma evolution under therapy reveals cell-intrinsic and cell-extrinsic therapeutic targets. Nat. Cancer.

[28] Koh L., Novera W., Lim S.W., Chong Y.K., Pang Q.Y., Low D., Ang B.T., Tang C. (2022). Integrative multi-omics approach to targeted therapy for glioblastoma. Pharmacol. Res..

[29] Heo Y.J., Hwa C., Lee G.H., Park J.M., An J.Y. (2021). Integrative Multi-Omics Approaches in Cancer Research: From Biological Networks to Clinical Subtypes. Mol. Cells.

[30] Ravi V.M., Will P., Kueckelhaus J., Sun N., Joseph K., Salié H., Vollmer L., Kuliesiute U., von Ehr J., Benotmane J.K. (2022). Spatially resolved multi-omics deciphers bidirectional tumor-host interdependence in glioblastoma. Cancer Cell.

[31] Joseph J.V., Blaavand M.S., Daubon T., Kruyt F.A., Thomsen M.K. (2021). Three-dimensional culture models to study glioblastoma—Current trends and future perspectives. Curr. Opin. Pharmacol..

[32] Mariappan A., Goranci-Buzhala G., Ricci-Vitiani L., Pallini R., Gopalakrishnan J. (2021). Trends and challenges in modeling glioma using 3D human brain organoids. Cell Death Differ..

[33] Zhang C.C., Jin M.Z., Zhao J.N., Chen J.X., Jin W.L. (2020). Organoid models of glioblastoma: Advances, applications and challenges. Am. J. Cancer Res..

[34] Bikfalvi A., da Costa C.A., Avril T., Barnier J.V., Bauchet L., Brisson L., Cartron P.F., Castel H., Chevet E., Chneiweiss H. (2023). Challenges in glioblastoma research: Focus on the tumor microenvironment. Trends Cancer.

[35] Dapash M., Hou D., Castro B., Lee-Chang C., Lesniak M.S. (2021). The Interplay between Glioblastoma and Its Microenvironment. Cells.

[36] Sharma P., Aaroe A., Liang J.Y., Puduvalli V.K. (2023). Tumor microenvironment in glioblastoma: Current and emerging concepts. Neuro-Oncol. Adv..

[37] Nakajima N., Nobusawa S., Nakata S., Nakada M., Yamazaki T., Matsumura N., Harada K., Matsuda H., Funata N., Nagai S. (2018). BRAF V600E, TERT promoter mutations and CDKN2A/B homozygous deletions are frequent in epithelioid glioblastomas: A histological and molecular analysis focusing on intratumoral heterogeneity. Brain Pathol..

[38] Phillips R.E., Soshnev A.A., Allis C.D. (2020). Epigenomic Reprogramming as a Driver of Malignant Glioma. Cancer Cell.

[39] Montella L., Cuomo M., Del Gaudio N., Buonaiuto M., Costabile D., Visconti R., Di Risi T., Vinciguerra R., Trio F., Ferraro S. (2023). Epigenetic alterations in glioblastomas: Diagnostic, prognostic and therapeutic relevance. Int. J. Cancer.

[40] Uddin M.S., Al Mamun A., Alghamdi B.S., Tewari D., Jeandet P., Sarwar M.S., Ashraf G.M. (2022). Epigenetics of glioblastoma multiforme: From molecular mechanisms to therapeutic approaches. Semin. Cancer Biol..

[41] Hegi M.E., Diserens A.C., Gorlia T., Hamou M.F., de Tribolet N., Weller M., Kros J.M., Hainfellner J.A., Mason W., Mariani L. (2005). MGMT gene silencing and benefit from temozolomide in glioblastoma. N. Engl. J. Med..

[42] Mansouri A., Hachem L.D., Mansouri S., Nassiri F., Laperriere N.J., Xia D., Lindeman N.I., Wen P.Y., Chakravarti A., Mehta M.P. (2019). MGMT promoter methylation status testing to guide therapy for glioblastoma: Refining the approach based on emerging evidence and current challenges. Neuro-Oncology.

[43] Della Monica R., Cuomo M., Buonaiuto M., Costabile D., Franca R.A., De Caro M.D., Catapano G., Chiariotti L., Visconti R. (2022). MGMT and Whole-Genome DNA Methylation Impacts on Diagnosis, Prognosis and Therapy of Glioblastoma Multiforme. Int. J. Mol. Sci..

[44] Noushmehr H., Weisenberger D.J., Diefes K., Phillips H.S., Pujara K., Berman B.P., Pan F., Pelloski C.E., Sulman E.P., Bhat K.P. (2010). Identification of a CpG island methylator phenotype that defines a distinct subgroup of glioma. Cancer Cell.

[45] Capper D., Jones D.T.W., Sill M., Hovestadt V., Schrimpf D., Sturm D., Koelsche C., Sahm F., Chavez L., Reuss D.E. (2018). DNA methylation-based classification of central nervous system tumours. Nature.

[46] Klughammer J., Kiesel B., Roetzer T., Fortelny N., Nemc A., Nenning K.H., Furtner J., Sheffield N.C., Datlinger P., Peter N. (2018). The DNA methylation landscape of glioblastoma disease progression shows extensive heterogeneity in time and space. Nat. Med..

[47] McClellan B.L., Haase S., Nunez F.J., Alghamri M.S., Dabaja A.A., Lowenstein P.R., Castro M.G. (2023). Impact of epigenetic reprogramming on antitumor immune responses in glioma. J. Clin. Investig..

[48] Romani M., Pistillo M.P., Banelli B. (2018). Epigenetic Targeting of Glioblastoma. Front. Oncol..

[49] Ghiaseddin A., Reardon D., Massey W., Mannerino A., Lipp E.S., Herndon J.E., Mcsherry F., Desjardins A., Randazzo D., Friedman H.S. (2018). Phase II Study of Bevacizumab and Vorinostat for Patients with Recurrent World Health Organization Grade 4 Malignant Glioma. Oncologist.

[50] Banasavadi-Siddegowda Y.K., Welker A.M., An M., Yang X.Z., Zhou W., Shi G.Q., Imitola J., Li C.L., Hsu S., Wang J. (2018). PRMT5 as a druggable target for glioblastoma therapy. Neuro-Oncology.

[51] Phillips H.S., Kharbanda S., Chen R.H., Forrest W.F., Soriano R.H., Wu T.D., Misra A., Nigro J.M., Colman H., Soroceanu L. (2006). Molecular subclasses of high-grade glioma predict prognosis, delineate a pattern of disease progression, and resemble stages in neurogenesis. Cancer Cell.

[52] Verhaak R.G.W., Hoadley K.A., Purdom E., Wang V., Qi Y., Wilkerson M.D., Miller C.R., Ding L., Golub T., Mesirov J.P. (2010). Integrated Genomic Analysis Identifies Clinically Relevant Subtypes of Glioblastoma Characterized by Abnormalities. Cancer Cell.

[53] Becker A.P., Sells B.E., Haque S.J., Chakravarti A. (2021). Tumor Heterogeneity in Glioblastomas: From Light Microscopy to Molecular Pathology. Cancers.

[54] Antunes A.R.P., Scheyltjens I., Duerinck J., Neyns B., Movahedi K., Van Ginderachter J.A. (2020). Understanding the glioblastoma immune microenvironment as basis for the development of new immunotherapeutic strategies. eLife.

[55] Di Nunno V., Franceschi E., Tosoni A., Gatto L., Bartolini S., Brandes A.A. (2022). Glioblastoma Microenvironment: From an Inviolable Defense to a Therapeutic Chance. Front. Oncol..

[56] Martínez A.H., Madurga R., García-Romero N., Ayuso-Sacido A. (2022). Unravelling glioblastoma heterogeneity by means of single-cell RNA sequencing. Cancer Lett..

[57] Patel A.P., Tirosh I., Trombetta J.J., Shalek A.K., Gillespie S.M., Wakimoto H., Cahill D.P., Nahed B.V., Curry W.T., Martuza R.L. (2014). Single-cell RNA-seq highlights intratumoral heterogeneity in primary glioblastoma. Science.

[58] Meyer M., Reimand J., Lan X.Y., Head R., Zhu X.M., Kushida M., Bayani J., Pressey J.C., Lionel A.C., Clarke I.D. (2015). Single cell-derived clonal analysis of human glioblastoma links functional and genomic heterogeneity. Proc. Natl. Acad. Sci. USA.

[59] Darmanis S., Sloan S.A., Croote D., Mignardi M., Chernikova S., Samghababi P., Zhang Y., Neff N., Kowarsky M., Caneda C. (2017). Single-Cell RNA-Seq Analysis of Infiltrating Neoplastic Cells at the Migrating Front of Human Glioblastoma. Cell Rep..

[60] Venteicher A.S., Tirosh I., Hebert C., Yizhak K., Neftel C., Filbin M.G., Hovestadt V., Escalante L.E., Shaw M.L., Rodman C. (2017). Decoupling genetics, lineages, and microenvironment in IDH-mutant gliomas by single-cell RNA-seq. Science.

[61] Meng Q.K., Zhang Y., Li G.Q., Li Y.N., Xie H.B., Chen X.J. (2021). New insights for precision treatment of glioblastoma from analysis of single-cell lncRNA expression. J. Cancer Res. Clin. Oncol..

[62] Wu H.B., Guo C.C., Wang C.Y., Xu J., Zheng S.Y., Duan J., Li Y.Y., Bai H.M., Xu Q.Y., Ning F.L. (2023). Single-cell RNA sequencing reveals tumor heterogeneity, microenvironment, and drug-resistance mechanisms of recurrent glioblastoma. Cancer Sci..

[63] Neftel C., Laffy J., Filbin M.G., Hara T., Shore M.E., Rahme G.J., Richman A.R., Silverbush D., Shaw M.L., Hebert C.M. (2019). An Integrative Model of Cellular States, Plasticity, and Genetics for Glioblastoma. Cell.

[64] Korshunov A., Schrimpf D., Ryzhova M., Sturm D., Chavez L., Hovestadt V., Sharma T., Habel A., Burford A., Jones C. (2017). H3-/IDH-wild type pediatric glioblastoma is comprised of molecularly and prognostically distinct subtypes with associated oncogenic drivers. Acta Neuropathol..

[65] Khabibov M., Garifullin A., Boumber Y., Khaddour K., Fernandez M., Khamitov F., Khalikova L., Kuznetsova N., Kit O., Kharin L. (2022). Signaling pathways and therapeutic approaches in glioblastoma multiforme (Review). Int. J. Oncol..

[66] Alexandru O., Horescu C., Sevastre A.S., Cioc C.E., Baloi C., Oprita A., Dricu A. (2020). Receptor tyrosine kinase targeting in glioblastoma: Performance, limitations and future approaches. Wspolczesna Onkol..

[67] Li X.M., Wu C.J., Chen N.C., Gu H.D., Yen A., Cao L., Wang E.H., Wang L. (2016). PI3K/Akt/mTOR signaling pathway and targeted therapy for glioblastoma. Oncotarget.

[68] Dewdney B., Jenkins M.R., Best S.A., Freytag S., Prasad K., Holst J., Endersby R., Johns T.G. (2023). From signalling pathways to targeted therapies: Unravelling glioblastoma’s secrets and harnessing two decades of progress. Signal Transduct. Target. Ther..

[69] Oprita A., Baloi S.C., Staicu G.A., Alexandru O., Tache D.E., Danoiu S., Micu E.S., Sevastre A.S. (2021). Updated Insights on EGFR Signaling Pathways in Glioma. Int. J. Mol. Sci..

[70] Qin A., Musket A., Musich P.R., Schweitzer J.B., Xie Q. (2021). Receptor tyrosine kinases as druggable targets in glioblastoma: Do signaling pathways matter?. Neuro-Oncol. Adv..

[71] Kim G., Ko Y.T. (2020). Small molecule tyrosine kinase inhibitors in glioblastoma. Arch. Pharm. Res..

[72] Tilak M., Holborn J., New L.A., Lalonde J., Jones N. (2021). Receptor Tyrosine Kinase Signaling and Targeting in Glioblastoma Multiforme. Int. J. Mol. Sci..

[73] Zhang Y., Dube C., Gibert M., Cruickshanks N., Wang B.M., Coughlan M., Yang Y.Z., Setiady I., Deveau C., Saoud K. (2018). The p53 Pathway in Glioblastoma. Cancers.

[74] Chkheidze R., Raisanen J., Gagan J., Richardson T.E., Pinho M.C., Raj K., Achilleos M., Slepicka C., White C.L., Evers B.M. (2021). Alterations in the RB Pathway With Inactivation of Characterize Glioblastomas With a Primitive Neuronal Component. J. Neuropathol. Exp. Neurol..

[75] Suwala A.K., Stichel D., Schrimpf D., Maas S.L.N., Sill M., Dohmen H., Banan R., Reinhardt A., Sievers P., Hinz F. (2021). Glioblastomas with primitive neuronal component harbor a distinct methylation and copy-number profile with inactivation of TP53, PTEN, and RB1. Acta Neuropathol..

[76] Tien A.C., Li J., Bao X., Derogatis A., Kim S., Mehta S., Sanai N. (2019). A Phase 0 Trial of Ribociclib in Recurrent Glioblastoma Patients Incorporating a Tumor Pharmacodynamic- and Pharmacokinetic-Guided Expansion Cohort. Clin. Cancer Res..

[77] Lee A., Arasaratnam M., Chan D.L.H., Khasraw M., Howell V.M., Wheeler H. (2020). Anti-epidermal growth factor receptor therapy for glioblastoma in adults. Cochrane Syst. Rev..

[78] Rodriguez S.M.B., Kamel A., Ciubotaru G.V., Onose G., Sevastre A.S., Sfredel V., Danoiu S., Dricu A., Tataranu L.G. (2023). An Overview of EGFR Mechanisms and Their Implications in Targeted Therapies for Glioblastoma. Int. J. Mol. Sci..

[79] Khan I., Waqas M., Shamim M.S. (2017). Prognostic significance of IDH 1 mutation in patients with glioblastoma multiforme. J. Pak. Med. Assoc..

[80] Han S., Liu Y., Cai S.R.J., Qian M.Y., Ding J.Y., Larion M., Gilbert M.R., Yang C.Z. (2020). IDH mutation in glioma: Molecular mechanisms and potential therapeutic targets. Br. J. Cancer.

[81] Butler M., Pongor L., Su Y.T., Xi L.Q., Raffeld M., Quezado M., Trepel J., Aldape K., Pommier Y., Wu J. (2020). MGMT Status as a Clinical Biomarker in Glioblastoma. Trends Cancer.

[82] Yan H., Parsons D.W., Jin G., McLendon R., Rasheed B.A., Yuan W., Kos I., Batinic-Haberle I., Jones S., Riggins G.J. (2009). IDH1 and IDH2 mutations in gliomas. N. Engl. J. Med..

[83] Brar H.K., Jose J., Wu Z.M., Sharma M. (2023). Tyrosine Kinase Inhibitors for Glioblastoma Multiforme: Challenges and Opportunities for Drug Delivery. Pharmaceutics.

[84] Aldaz P., Arozarena I. (2021). Tyrosine Kinase Inhib. Adult Glioblastoma: (Un)Closed Chapter?. Cancers.

[85] Weller M., Butowski N., Tran D.D., Recht L.D., Lim M., Hirte H., Ashby L., Mechtler L., Goldlust S.A., Iwamoto F. (2017). Rindopepimut with temozolomide for patients with newly diagnosed, EGFRvIII-expressing glioblastoma (ACT IV): A randomised, double-blind, international phase 3 trial. Lancet Oncol..

[86] Taylor J.W., Parikh M., Phillips J.J., James C.D., Molinaro A.M., Butowski N.A., Clarke J.L., Oberheim-Bush N.A., Chang S.M., Berger M.S. (2018). Phase-2 trial of palbociclib in adult patients with recurrent RB1-positive glioblastoma. J. Neuro-Oncol..

[87] Lee E.Q., Trippa L., Fell G., Rahman R., Arrillaga-Romany I., Touat M., Drappatz J., Welch M.R., Galanis E., Ahluwalia M.S. (2021). Preliminary results of the abemaciclib arm in the Individualized Screening Trial of Innovative Glioblastoma Therapy (INSIGhT): A phase II platform trial using Bayesian adaptive randomization. J. Clin. Oncol..

[88] Sepúlveda-Sánchez J.M., Gil-Gil M., Alonso-García M., Salgado M.A.V., Vicente E., Barroso C.M., Sánchez A.R., Durán G., De Las Peñas R., Muñoz-Langa J. (2020). Phase II Trial of Palbociclib in Recurrent Retinoblastoma-Positive Anaplastic Oligodendroglioma: A Study from the Spanish Group for Research in Neuro-Oncology (GEINO). Target. Oncol..

[89] van den Bent M.J., Klein M., Smits M., Reijneveld J.C., French P.J., Clement P., de Vos F.Y.F., Wick A., Mulholland P.J., Taphoorn M.J.B. (2018). Bevacizumab and temozolomide in patients with first recurrence of WHO grade II and III glioma, without 1p/19q co-deletion (TAVAREC): A randomised controlled phase 2 EORTC trial. Lancet Oncol..

[90] Wick W., Gorlia T., Bendszus M., Taphoorn M., Sahm F., Harting I., Brandes A.A., Taal W., Domont J., Idbaih A. (2017). Lomustine and Bevacizumab in Progressive Glioblastoma. N. Engl. J. Med..

[91] Reardon D.A., Brandes A.A., Omuro A., Mulholland P., Lim M., Wick A., Baehring J., Ahluwalia M.S., Roth P., Bähr O. (2020). Effect of Nivolumab vs Bevacizumab in Patients With Recurrent Glioblastoma The CheckMate 143 Phase 3 Randomized Clinical Trial. JAMA Oncol..

[92] Weller M., Lim M., Idbaih A., Steinbach J., Finocchiaro G., Raval R., Ashby L., Ansstas G., Baehring J., Taylor J. (2021). A Randomized Phase 3 Study of Nivolumab or Placebo Combined with Radiotherapy Plus Temozolomide in Patients with Newly Diagnosed Glioblastoma with Methylated Mgmt Promoter: Checkmate 548. Neuro-Oncology.

[93] Sampson J.H., Omuro A.M.P., Preusser M., Lim M., Butowski N.A., Cloughesy T.F., Strauss L.C., Latek R.R., Paliwal P., Weller M. (2016). A randomized, phase 3, open-label study of nivolumab versus temozolomide (TMZ) in combination with radiotherapy (RT) in adult patients (pts) with newly diagnosed, O-6-methylguanine DNA methyltransferase (MGMT)-unmethylated glioblastoma (GBM): CheckMate-498. J. Clin. Oncol..

[94] Nayak L., Molinaro A.M., Peters K., Clarke J.L., Jordan J.T., de Groot J., Nghiemphu L., Kaley T., Colman H., McCluskey C. (2021). Randomized Phase II and Biomarker Study of Pembrolizumab plus Bevacizumab versus Pembrolizumab Alone for Patients with Recurrent Glioblastoma. Clin. Cancer Res..

[95] van den Bent M., Eoli M., Sepulveda J.M., Smits M., Walenkamp A., Frenel J.S., Franceschi E., Clement P.M., Chinot O., de Vos F.Y.F.L. (2020). INTELLANCE 2/EORTC 1410 randomized phase II study of Depatux-M alone and with temozolomide vs temozolomide or lomustine in recurrent EGFR amplified glioblastoma. Neuro-Oncology.

[96] Bagley S.J., Desai A.S., Linette G.P., June C.H., O’Rourke D.M. (2018). CAR T-cell therapy for glioblastoma: Recent clinical advances and future challenges. Neuro-Oncology.

[97] Lim M., Xia Y.X., Bettegowda C., Weller M. (2018). Current state of immunotherapy for glioblastoma. Nat. Rev. Clin. Oncol..

[98] Xu S.C., Tang L., Li X.Z., Fan F., Liu Z.X. (2020). Immunotherapy for glioma: Current management and future application. Cancer Lett..

[99] Mahmoud A.B., Ajina R., Aref S., Darwish M., Alsayb M., Taher M., AlSharif S.A., Hashem A.M., Alkayyal A.A. (2022). Advances in immunotherapy for glioblastoma multiforme. Front. Immunol..

